# Evaluation of Portable, Low-Cost Autorefractor in School Students with Limited Eye Care Access in Northeastern Brazil

**DOI:** 10.3390/vision9010017

**Published:** 2025-02-27

**Authors:** Francisco Carlos de Castro Neto, Ricardo Noguera Louzada, Daniel Oliveira Dantas, Dillan Cunha Amaral, Claudio do Carmo Chaves Filho, Milton Ruiz Alves

**Affiliations:** 1 Division of Ophthalmology, Faculty of Medicine, University of São Paulo, São Paulo 05403-000, SP, Brazil; carlos.castro.neto@gmail.com (F.C.d.C.N.); claudiochavesf@ufam.edu.br (C.d.C.C.F.); miltonruizcbo@gmail.com (M.R.A.); 2 School of Medicine, Estacio Medical School of Juazeiro, Juazeiro 48924-999, BA, Brazil; 3 Department of Computer Science, Federal University of Sergipe, São Cristóvão 49107-230, SE, Brazil; dantasu@gmail.com; 4 Ophthalmology, Faculty of Medicine, Federal University of Rio de Janeiro, Rio de Janeiro 21941-617, RJ, Brazil; dillanamaral@ufrj.br; 5 Department of Clinical Surgery, Faculty of Medicine, Federal University of Amazonas, Manaus 69020-160, AM, Brazil

**Keywords:** refraction, ocular, equipment design, comparative study

## Abstract

This study compared the refraction measurements of the ClickCheck™ device (CCD), Topcon KR-8000, and subjective clinical refractometry (SCR) in Brazilian public school students with limited access to eye care. Eighty-seven eyes of healthy students aged 7 to 17 underwent refractometry using the CCD, Topcon KR-8000, and SCR methods under cycloplegia, with only the right-eye data analyzed. For comparison, the measurements were converted into spherical equivalents (SEs) and vector magnitudes. The mean SE difference between CCD and Topcon KR-8000 was −0.27 ± 0.58 (*p* < 0.0001), while the vector magnitudes at the 90° and 135° meridians were −0.23 ± 0.55 (*p* = 0.0001) and +0.04 ± 0.47 (*p* = 0.2246), respectively, demonstrating no clinical relevance. Similarly, the mean SE difference between CCD and SCR was −0.18 ± 0.58 (*p* = 0.065), with vector magnitudes of −0.20 ± 0.50 (*p* = 0.0003) at 90° and +0.03 ± 0.46 (*p* = 0.3730) at 135°, also lacking clinical relevance. Despite statistically significant differences between the methods, the findings confirm strong agreement, validating CCD as an effective refractive assessment tool for children in low-resource settings. These methods can enhance access to refraction services in underserved populations.

## 1. Introduction

Uncorrected refractive error (URE) is a significant public health issue, particularly among children, and it significantly impacts academic performance and overall development [1,2,3,4,5,6,7,8,9,10]. This is evidenced by its inclusion in the priority areas of Vision 2020: The Right to Sight Program, a global initiative led by a consortium of non-governmental organizations and the World Health Organization [6,7].

Implementing preventive eye health measures for elementary and middle school students with restricted access to eye care should include early case detection, eye examination, refraction, eyeglass dispensing, and follow-up to ensure prescription compliance [11,12]. The Brazilian Council of Ophthalmology estimates that approximately 20% of schoolchildren have some eye condition, including refractive errors, amblyopia, and strabismus [13]. A population-based study in Brazil found that 13.8% of the participants had URE, with 4.6% experiencing optically reversible low vision and 1.8% facing reversible blindness through optical correction [14]. In regions with limited access to ophthalmic care, the prevalence of uncorrected refractive errors is uncertain and may range from 3.5% to 14.11% [15,16,17]. Therefore, it highlights the need for new actions to control the growing demand flow and expand students’ access to Eye Health Services [13]. 

One of the ways to broaden ophthalmological care for schoolchildren includes incorporating innovative technologies into this process [18,19]. There is a global effort in this regard, particularly in developing simple and inexpensive devices for the self-detection of myopia, such as smartphone apps [20]. Performing ocular refraction evaluations at school using computerized refractors is difficult because of these devices’ high cost and need for portability. Low-cost, portable autorefractors like the CCD (Essilor International S.A., Charenton-le-Pont, France) enable school-based refractive error screening, especially in underserved regions [14,15]. Its plastic design allows easy sanitization by wiping the tube and eyepiece [14]. 

This study compared the accuracy of refraction measurements taken with the CCD, Topcon KR-8000, and subjective clinical refractometry (SCR) in elementary and middle school students aged 7 to 17 with limited access to eye care in Brazil’s northeastern region.

## 2. Materials and Methods

Following approval of the study protocol by the Ethics Committee for Research Analysis of the University Hospital of the Federal University of Vale do São Francisco (HU/UNIVASF), CAAE No. 7099753.8.0000.0282, informed consent was obtained from all the students’ parents or legal representatives. The study population consisted of eighty-seven schoolchildren enrolled in elementary and middle school in the municipality of Juazeiro, Bahia, aged 7 to 17 years. Data were collected on the age, date of birth, gender, and ophthalmological findings.

At the time of the ophthalmological evaluation, the students identified with visual acuity (VA) without correction ≤ 20/40 (0.5) who did not present any active condition of an allergic, inflammatory, or infectious nature on the ocular surface or adnexa were referred for a complete ophthalmological evaluation.

The ophthalmological assessments followed this sequence: (1) VA testing using a Snellen chart at 5 meters without optical correction, (2) three refractive measurements with the CCD device without cycloplegia, (3) three CCD measurements under cycloplegia, (4) three refractive measurements using the Topcon KR-8000 under cycloplegia, (5) SCR under cycloplegia with a Greens refractor, (6) VA assessment with correction, and (7) slit-lamp biomicroscopy and fundoscopy. The CCD autorefractor utilized in this study was supplied at no cost by Essilor (Rio de Janeiro, Brazil).

Cycloplegia was achieved by instilling a drop of 1% cyclopentolate (cyclopentolate 10 mg/mL, Allergan–AbbVie, Inc., North Chicago, IL, USA),which was complemented after 5 minutes with a drop of 1% tropicamide (Mydriacyl 1%, Novartis, Basel, Switzerland). A refraction examination was performed 30 minutes after the first installation.

### 2.1. Sample Size Calculation

According to Joseph et al., the standard deviation of the spherical corrections obtained with subjective refraction was 1.85, and, with the ClickCheck, it was 2.20 [21,22]. A power analysis indicated that a sample size of 72 individuals would be enough for a single-sided test to detect a difference of 1 diopter between both groups with 0.9 power and a significance level of 0.05.

### 2.2. Statistical Analysis

Refractive measurements of the right eye were used only to evaluate the accuracy of the refraction of the CCD. The mean refractive error measurement results were compared without and with cycloplegia from CCD and the Topcon KRT-8000 autorefractor and SCR. In the refractive error analysis, the spherical component was measured in spherical diopters, while the cylindrical component was recorded in cylindrical diopters, with the cylinder’s principal axis expressed in degrees. To facilitate statistical evaluation, values were transformed into spherical equivalents (SEs), calculated as the spherical value plus half of the astigmatism value. Additionally, both spherical and cylindrical components were transformed into power vectors using the Naeser equation: MV90 = m (sin 2a − cos 2a), where MV90 represents the magnitude vector along the 90° axis: m denotes astigmatism in diopters: and a refers to the astigmatism meridian in degrees, accounting for both vertical and horizontal refraction components.

The equation MV 135 = m(sen2(a − 45) − cos2(a − 45) allows the calculation of the difference between diopter components projected on the 135° axis and the 45° axis. The MV components were divided in half to maintain the SE format. Statistical calculations and plots were completed using the software R version 4.4.1, with the libraries pwr version 1.3.0, car version 3.1.2, and scatterplot3d version 0.3.44 [23]. The univariate analysis was performed as follows. The SE value differences were determined considering the SE values of the CCD refraction minus the SE values of the Topcon KR-8000 refraction and the SE values of the CCD refraction minus the SE values of the SCR. The same procedure was applied to MV90 and MV135, respectively. A positive difference indicated that CCD overestimated the corresponding value.

Comparisons between measurements were performed using a Wilcoxon signed-rank test. Bivariate and trivariate analyses were performed using the Hoteling’s test. The level of statistical significance was set at 5% (*p* < 0.05).

## 3. Results

The visual screening performed at the school identified 87 students with uncorrected VA ≤ 20/40 (0.5). The mean age was 14.48 ± 2.66 years (7 to 17 years), with 35 (40.23%) males and 52 (59.77%) females. These students underwent refractometry and achieved VA 1.0 with the best correction. 

Table 1 shows the differences between the Topcon KR-8000 and SCR refraction values obtained under cycloplegia. The data showed that Topcon KR-8000 tended to overestimate the spherical and cylindrical powers, although these differences were less than 0.10D and had no clinical relevance.

Table 2 shows the differences between the CCD and Topcon KR-8000 refraction values obtained under cycloplegia. The CCD produced the following mean ± SD: SE was −0.27 ± 0.58 (*p* < 0.0001); MV90 was −0.23 ± 0.55 (*p* = 0.0001); and MV135 was +0.04 ± 0.47 (*p* = 0.2246). The data showed that CCD tended to underestimate both the spherical and cylindrical powers, although these differences were less than 0.50D and had no clinical relevance.

Table 3 shows the differences between the refraction values obtained by CCD and SCR under cycloplegia. The CCD produced the following mean ± SD: SE was −0.18 ± 0.58 (*p* = 0.0065), MV90 was −0.21 ± 2.87 (*p* = 0.4142), and MV135 was +0.03 ± 0.46 (*p* = 0.2529). The data showed that CCD tended to overestimate the Spherical and Cylindrical powers, although these differences were less than 0.25D and had no clinical relevance. 

**Figure 1:**
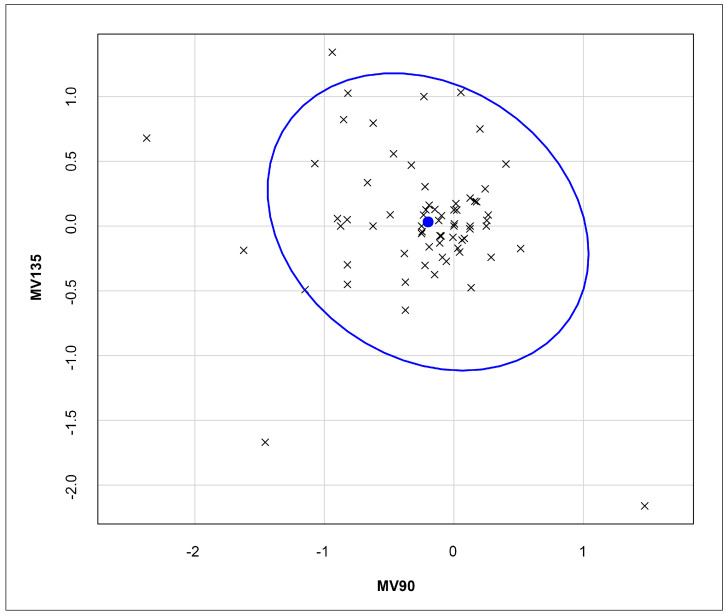
A bivariate analysis evaluating the influence of astigmatism on the differences between the refraction values obtained by ClickCheck^TM^ and SCR, both with cycloplegia. Assuming a bivariate normal distribution, the blue ellipsis indicates the region that contains 95% of the data points. The blue dot indicates the mean value.

A bivariate analysis was performed to evaluate the influence of astigmatism on the differences between the refraction values obtained by CCD and SCR, both under cycloplegia (Figure 1). The portion within the ellipse represents 95% of the sample, demonstrating statistical significance when comparing these two studied parameters related to astigmatism: MV90 and MV135.

**Figure 2:**
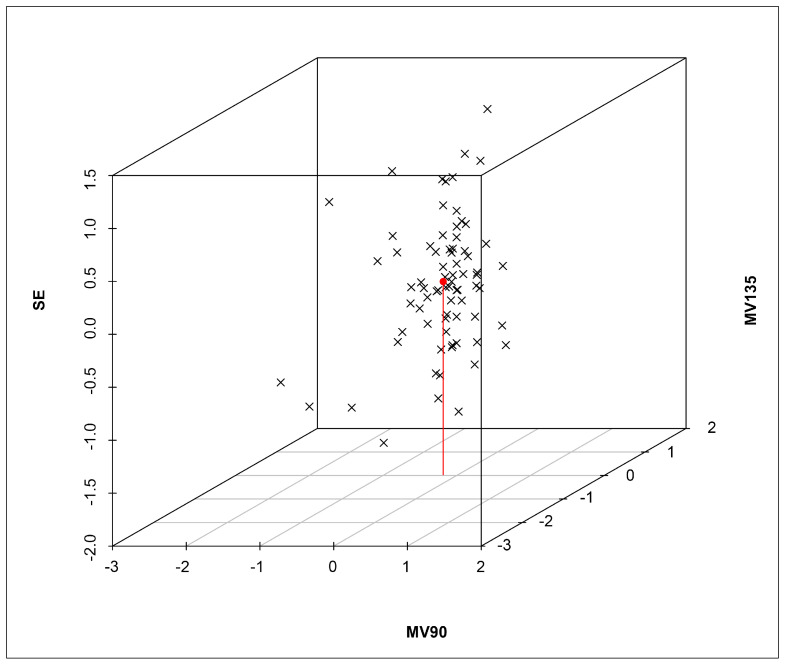
A trivariate analysis and a 3D plot assessed the relationships among SE, MV90, and MV135 and their influence on differences between the right-eye refraction values obtained under cycloplegia using ClickCheck^TM^ and SCR. The red dot indicates the mean value and the red line indicates its projection on the plane MV90 versus MV135.

A trivariate analysis was conducted, utilizing a 3D plot to examine the relationships among SE, MV90, and MV135 and their impact on the differences in right-eye refraction measurements under cycloplegia using CCD and SCR (Figure 2).

The conversion of refraction into vector values for the conventional form revealed that, on average, the difference between the CCD measurement and the SCR, both under cycloplegia, was +0.0271DE with −0.4048 × 85.42, with an SE of +0.1753D for each student’s right eye.

## 4. Discussion

URE is a severe public health problem that needs to be addressed globally [3]. Ophthalmology in Brazil has yet to be incorporated into SUS Primary Care, which partly explains the difficulty of the public service in offering ophthalmological consultations. These factors highlight the need for innovative strategies for ocular refraction assessment and eyeglass prescription [18,19]. The results of this study demonstrate the feasibility of alternative methods for identifying refractive errors and correcting them with glasses. In this context, CCD, an inexpensive and portable autorefractor that can accurately measure spherical refractive error, can contribute to diagnosing and treating URE, especially in low-income countries. The effectiveness of the CCD in measuring the refractive spherical error has already been demonstrated in populations aged 7 to 70 [21]. For instance, Joseph et al., involving 1079 participants, reported an intraclass correlation coefficient of 0.940 (95% CI: 0.933 to 0.947) between ClickCheck™ and subjective refraction for spherical measurements [21]. However, it has shown limitations in obtaining accurate astigmatic power and axis data.

The CCD strongly agreed with Topcon KR-8000, with mean refractive error differences below 0.50D, demonstrating no clinical relevance (Table 2) [21]. The gold-standard method for measuring refractive errors in children is cycloplegic retinoscopy; however, tabletop autorefractometers demonstrate a strong correlation with retinoscopy results, showing excellent agreement for spherical, cylindrical, and SE values obtained under cycloplegia [24,25]. A key advantage of the CCD is that it is a lightweight, easy-to-use, inexpensive autorefractor with great portability, enabling students to be identified in visual screening and subjected to refractometry at the school itself, thus reducing absenteeism.

Furthermore, univariate analysis of the differences between the values of RE refraction measured by CCD and SCR, both under cycloplegia, showed that the mean difference in refractive error in SE and the means of the differences in the vector magnitude in the 900 and 1350 meridians were less than 0.25D and were considered to have no clinical relevance (Table 3) [21].

The bivariate analysis (Figure 1) shows a strong correlation between MV90 and MV135 for CCD and SCR, with most data points within the 95% confidence ellipse. This suggests that, while minor variations exist, the CCD’s ability to assess astigmatism is statistically comparable with that of the SCR method. However, the spread of data points within the ellipse may indicate some degree of variability, meaning that the CCD could slightly underestimate or overestimate specific astigmatic components. The trivariate analysis (Figure 2) reinforced this agreement across SE, MV90, and MV135. Although slight variations in astigmatic measurements (−0.4048 × 85.42) were observed, these differences remained within an acceptable clinical range. The low SE variation (+0.1753D) further reinforced the practicality of CCD for school-based screenings, as the minor deviations would not significantly impact corrective prescriptions.

An essential limitation of this study was the small sample size. Another limitation of this study is that the CCD has a refractive range (spherical correction −11.00D to +8.00D and cylindrical correction up to 4.00DC) that covers most of the population. Therefore, there is always the possibility of high refractive errors being left out. However, this range of refractive corrections was sufficient for all the schoolchildren included in this study. Further studies evaluating CCD performance in broader age groups would be valuable in assessing its applicability beyond school settings. Using the CCD requires an understanding of its operation and application, which may pose challenges for children under seven years old, whereas it is easier for adolescents and adults. It is important to note that many portable refractors, although much more expensive than the CCD (USD 64.00), are also easy to use and use innovative technological approaches, presenting lower, comparable, or higher accuracy levels than the CCD [18,24,25,26,27,28,29,30].

In conclusion, the CCD strongly agreed with the Topcon KR-8000 autorefractor and SCR in measuring refractive errors among schoolchildren aged 7 to 17, all under cycloplegia. The CCD’s advantages over conventional autorefractors include its low cost, portability, and ease of use with minimal training. This study shows that these approaches can improve refraction services in low-resource settings.

## Figures and Tables

**Table 1:** Univariate analysis of differences between right-eye refraction values obtained under cycloplegia by Topcon KR-8000 and subjective clinical refractometry in a sample of eighty-seven elementary and middle school schoolchildren.

	Variables	Average	SD ^1^	*p*-Value
Topcon KR-8000* − SCR*	SE ^2^	0.09	0.41	0.0090
	MV90 ^3^	0.03	0.24	0.1108
	MV135 ^4^	−0.01	0.24	0.3233

^1^ SD: standard deviation; ^2^ SE: spherical equivalent; ^3^ MV90: magnitude vector on the 90° axis; ^4^ MV135: difference between diopter components projected on the 135° axis and the 45° axis; Topcon KR-8000* − SCR*: difference between refraction values obtained by Topcon KR-8000 refractometry and subjective clinical refractometry, both under cycloplegia.

**Table 2:** Univariate analysis of differences between right-eye refraction values obtained under cycloplegia by ClickCheck^TM^ and Topcon KR-8000 in a sample of eighty-seven elementary and middle school schoolchildren.

	Variables	Average	SD ^1^	*p*-Value
ClicCheck^TM^ − Topcon KR-8000*	SE ^2^	−0.27	0.58	<0.0001
	MV90 ^3^	−0.23	0.55	0.0045
	MV135 ^4^	+0.04	0.47	0.2246

^1^ SD: standard deviation; ^2^ SE: spherical equivalent; ^3^ MV90: magnitude vector on the 90° axis; ^4^ MV135: difference between diopter components projected on the 135° axis and the 45° axis; * ClickCheckTM − Topcon KR-8000: difference between refraction values obtained by ClickCheck^TM^ and Topcon KR-8000 refractometry, both under cycloplegia.

**Table 3:** Univariate analysis of differences between right-eye refraction values obtained under cycloplegia by ClickCheck^TM^ and subjective clinical refractometry in a sample of eighty-seven elementary and middle school schoolchildren.

	Variables	Average	SD ^1^	*p*-Value
ClickCheck^TM^ − SCR*	SE ^2^	−0.18	0.58	0.0065
	MV90 ^3^	−0.20	0.50	0.0003
	MV135 ^4^	+0.03	0.46	0.3730

^1^ SD: standard deviation; ^2^ SE: spherical equivalent; ^3^ MV90: magnitude vector on the 90° axis; ^4^ MV135: difference between diopter components projected on the 135° axis and the 45° axis; ClickCheck^TM^ − SCR*: the difference between refraction values obtained by ClickCheck^TM^ and subjective clinical refractometry, both under cycloplegia.

## Data Availability

The original contributions presented in this study are included in the article. Further inquiries can be directed to the corresponding author.
